# Harm Reduction: A Misnomer

**DOI:** 10.1007/s10728-020-00413-x

**Published:** 2020-11-05

**Authors:** Nicholas B. King

**Affiliations:** Biomedical Ethics Unit, McGill University, Montreal, Canada

**Keywords:** Harm reduction, Philosophy, Ethics, Utilitarianism, Justice

## Abstract

‘Harm reduction’ programs are usually justified on the utilitarian grounds that they aim to reduce the net harms of a behavior. In this paper, I contend that (1) the *historical genesis* of harm reduction programs, and the crucial *moral imperative* that distinguishes these programs from other interventions and policies, are not utilitarian; (2) the *practical implementation* of harm reduction programs is not, and probably cannot be, utilitarian; and (3) the *continued justification* of harm reduction on utilitarian grounds is untenable and may itself cause harm. Promoting harm reduction programs as utilitarian in the public arena disregards their deeper prioritarian impulses. ‘Harm reduction’ is a misnomer, and the name should be abandoned sooner rather than later.

## Introduction

Given the foregrounding of the words ‘harm’ (which implies an explicit assessment of harms and an implicit assessment of benefits) and ‘reduction’ (which implies a demonstrated decrease in harms), it is unsurprising that ‘harm reduction’ is generally considered a utilitarian approach to public health. Unsurprising, but wrong. In this paper, I contend that (1) the *historical genesis* of harm reduction programs, and the crucial *moral imperative* that distinguishes these programs from other interventions and policies, are not utilitarian; (2) the *practical implementation* of harm reduction programs is not, and probably cannot be, fully utilitarian; and (3) the *continued justification* of harm reduction on utilitarian grounds is untenable and may itself cause harm. Harm reduction programs are not utilitarian, they are rarely implemented in a spirit of utilitarianism, they should not be promoted or defended as such, and the extension of ‘harm reduction’ into new arenas under the banner of utilitarianism is misguided. For most of the programs that bear the name, ‘harm reduction’ is a potentially damaging misnomer.

### Defining Harm Reduction Programs as Utilitarian

Precise definitions of ‘harm reduction’ are hard to come by, and the term has been subject to robust debate. Programs bearing the name encompass a variety of actors, practices, and goals [4, 11], and attempts to define the term draw on multiple histories and claim compatibility with several philosophical positions. For example, Newcombe argues that harm reduction “has its main roots in the scientific public health model, with deeper roots in humanitarianism and libertarianism” [17], while the Harm Reduction International website contends that “harm reduction is grounded in justice and human rights.” Nevertheless, these are minority positions, and harm reduction is almost universally defined by two contrasts. The first, as Holland notes, is practical:

The key is to distinguish two responses to an unhealthy activity or risky health behavior. One response is to try to get people to stop behaving in that way, i.e., to try to get them to give up the activity in question. Policies with this intention are usually referred to as abstinence policies…The other response is to accept that, for whatever reason, it is unrealistic to expect people to give up the activity in question so it is better to allow it and try to reduce its harmful effects. Policies based on this response are known as ‘harm reduction’ [10].

There is some debate over whether harm reduction can ultimately be compatible with abstinence. Some claim that harm reduction should be sharply distinguished from abstinence programs [22]; others argue for including abstinence-oriented efforts within the purview of harm reduction [15]; still others chart a middle ground by stipulating that abstinence must be subsidiary to the primary goal of reducing harms [12]. All agree, however, that the philosophy of harm reduction presents a stark alternative to, and should be clearly demarcated from, abstinence-based programs.

Closely affiliated with this practical distinction between prohibition and acceptance is a conceptual one. As Christie et al. note, harm reduction is generally characterized as a consequentialist or utilitarian response to prohibitionist approaches that identify particular behaviors as wrong and seek to eliminate them:

implicit in the reasoning of harm reduction advocates is a Utilitarian argument, which holds that the key guideline in ethics is that if negative consequences can be avoided they should be avoided. Abstinence advocates, however, generally seem to employ a Deontological ethic, which maintains that the moral worth of one’s actions has nothing to do with the consequences of those actions but, rather, is determined by the intention of the actors [4].

Similarly, Wodak defines harm reduction as “a consequentialist approach to drug policy, emphasizing the importance of outcomes rather than the policy intent,” and argues that the debate between its advocates and its critics “is essentially a conflict between ‘consequentialists’, more concerned to evaluate interventions by considering their impact, while the ‘non-consequentialist’ critics of harm reduction prefer to evaluate interventions by considering their moral worth” [24]. Zajdow observes that “the utilitarian nature of harm minimization is seen in its proponents’ references to the necessity for evidence of programme effectiveness, its requirement for ‘a demonstration that harm reduction has occurred in an overall sense’” [25]. Even when “consequentialism” or “utilitarianism” are not explicitly cited as philosophical touchstones, reducing harmful consequences and conducting some analysis of costs and benefits are seen to play a central role, as in Riley et al.’s overview: “Harm reduction is a public-health approach to dealing with drug-related issues that places first priority on reducing the negative consequences of drug use rather than on eliminating drug use or ensuring abstinence,” in which “some pragmatic process of identifying, measuring, and assessing the relative importance of drug-related problems, their associated harms, and costs/benefits of intervention is carried out in order to focus resources on priority issues” [20].

The centrality of consequences to the definition of harm reduction is often mobilized as an indication of its pragmatism and value-neutrality, and a contrast to moralizing abstinence-based approaches [1, 5, 15, 17]. Thus, for example, Des Jarlais emphasizes “realistic pragmatism” as the “core value” of harm reduction: “drug policies must be pragmatic. They must be assessed on their actual consequences, not on whether they symbolically send the right, or wrong, or mixed messages” [5]. Keane similarly observes that “in opposition to strategies based on ‘arbitrary moralism’, the philosophy of harm reduction is generally assumed to promote rationality, pragmatism and utilitarianism in the development of drug interventions. Because the primary aim is reduction of harm rather than the attainment of ideals such as ‘a drug-free nation’, strategies can theoretically be assessed through an objective calculation of consequences, both costs and benefits” [11]. However, as Keane perceptively notes, harm reduction’s apparent self-evidence may ultimately reflect the dominance of consequentialism in the countries where it is practiced, rather than a true value neutrality [11].

An additional indication of the importance of utilitarianism within contemporary definitions of harm reduction can be found in the accounts of harm reduction’s critics. Supporters of abstinence-based programs openly reject consequentialist logic, in favor of deontologically-flavored arguments that drug use and other behaviors are categorically wrong [13]. More recent critics embrace some aspects of the implementation of harm reduction programs, but take issue with their consequentialist justification [8, 18, 23]. For example, Miller argues that “the notion of harm reduction as being based on the idea of a cost–benefit analysis is congruent with contemporary middle-class values (particularly economic rationalism)…the harm-minimization approach is in fact tailor-made to suit the current dominant ideology of economic rationalism, and does little to challenge many of the structural inequalities that, in the very least, contribute to problematic drug use” [16].

### Not (Just) About Consequences: Harm Reduction in Practice

While some form of utilitarian sensibility is almost universally *cited* as a core philosophical principle, when one looks more closely at the history and implementation of harm reduction, it is much harder to find evidence of utilitarianism as a moral imperative or guide to practice.

The history of harm reduction is complex and multifaceted, and is characterized by “a historic tension between those who see harm reduction primarily as a medical means of promoting health and mitigating the harm to individual, and a more activist group who see it as a platform for broader and more structural social change” [21]. While some early ‘top down,’ state-run programs embraced consequentialist justifications, its most passionate supporters have often advocated something rather different: community-based, ‘bottom-up’ approaches with political and moral commitments to remedy structural injustices and combat the oppression of marginalized groups such as injection drug users. Many of these advocates have uneasily embraced or outright rejected utilitarian justifications [21]. Indeed, some recent critics contend that the language of utilitarianism has alienated harm reduction from its true historical roots in humanitarianism and human rights [8], contributed to the erasure of its “explicitly radical, structurally-focused founding political philosophy” [23], and has contributed to its transformation “into a conservative movement, an apology for the past” [21] that mitigates the worst consequences of but ultimately preserves dysfunctional policies like the war on drugs.

On a conceptual level, it is difficult to see how utilitarianism and concern for consequences could possibly be a point that distinguishes ‘harm reduction’ from almost any other health or social policy [13]. In theory *anything* that could potentially cause harms, and whose harms could be reduced, can be or already is subject to a ‘harm reduction’ program. Speed limits reduce the harms of driving. Seat belts and motorcycle helmets reduce the harms of traffic accidents. HPV vaccination programs reduce the harms of sexual behavior. Food regulations reduce the harms of eating and drinking. Social security reduces the harms of growing old. Maintaining a strong military reduces the harms of living in a world with antagonistic neighbors. Many possible policies could also be thought of in terms of harm reduction: Putting statins in the water could reduce the harms of poor diets, sedentary lifestyles, and possibly air pollution. Dispensing anti-depressants and mood stabilizers to employees could reduce the harms of stressful occupations. Conducting a pre-emptive strike on military bases within a hostile neighbor’s territory could reduce the harms of future disputes. One could plausibly make the case that *any* health or social policy that reduces the negative impact of a human behavior could be considered a ‘harm reduction’ program. But in practice this is not the case, and few if any of the interventions listed above are explicitly classified as such.

Most interventions reduce harm. What characterizes actual ‘harm reduction’ programs is that they reduce particular sorts of harm: either harms that result from *behaviors* that are illegal, socially unacceptable, or widely stigmatized; or harms to *individuals or social groups* who are stigmatized, marginalized, or generally worse-off. This fact—that the objects of harm reduction programs are chosen according to value-laden criteria, and not on the basis of dispassionate calculations of overall benefits and costs—is the first clue that the moral imperative behind harm reduction programs is anything but utilitarian. Programs do not provide clean needles and supervised injection sites (SIS) to injectable drug users, or publicly-funded ‘wet houses’ to alcoholics, because they have conducted a cost–benefit analysis and determined that these interventions would be an efficient use of resources, and we can certainly think of more efficient public health uses for these resources. If utilitarianism is the impetus behind these programs, it is a form of utilitarianism that is so heavily constrained or poorly conceived as to barely deserve the name.

The fact that harm reduction programs explicitly benefit the worst off is the clearest clue that their ultimate impetus is not utilitarian. They don’t simply ‘reduce harms’ on a population basis, they address the suffering of specific kinds of people. Indeed, if harm reduction programs were subject to a rigorous cost–benefit assessment—say, by comparing them to other interventions that would cost the same amount of money—they would likely fare poorly. This is not a bug of harm reduction programs, it is a feature, because what these interventions do that distinguishes them from other interventions is to address the suffering of the worst-off. Harm reduction is driven by compassion and a de facto prioritarian sense of justice, not a utilitarian calculus.

One might reasonably contend that, even if the initial spirit of harm reduction was not utilitarian, its implementation surely is. Regardless of whether particular behaviors or groups are targeted, in practice these programs intend to, and actually do, reduce harms without making any sort of judgment about the morality of particular behaviors or individuals, which surely qualifies as the sort of value-neutral utilitarianism central to many of the definitions presented in the first section.

This is wrong on two counts. First, harm reduction programs are selective about the exact harms that they reduce, and this selectivity poses problems for a utilitarian defense. SIS programs reduce overdose-related mortality, and transmission of communicable diseases through needle-sharing. They do not, however, reduce many of the other harms associated with illicit drug use, including the negative aspects of addiction itself; indeed, a successful abstinence program would arguably do a better job at harm reduction than any extant harm reduction program. If we are to be truly utilitarian about our assessment of these programs, it is worth asking whether, on balance, the harm reduced by preventing an overdose death actually outweighs the harms of continuing to use illegal drugs, living with an addiction, and causing continued suffering to those who are addicted and their families and caregivers.

Second, harm reduction proponents routinely fail to fully account for *all* the harms associated with particular behaviors [13, 20]. Harm reduction programs rarely ask “what is the most efficient and effective way to, on balance, reduce the harms associated with particular behaviors,” appropriately weigh all the costs and benefits associated with different interventions, and then settle upon, say, supervised injection sites as the answer. Instead, harm reduction programs accept that a harmful behavior will occur, identify specific harms that could be reduced with a given intervention, and proceed to reduce the likelihood of those harms occurring. In some cases, some potential harmful effects of a program are accounted for, though usually only those that can be empirically demonstrated to be minimal or nonexistent. But many other harms are ignored. The cost/benefit calculus around harm reduction is always to some extent rigged. Indeed, some have argued that the cost/benefit calculus is rigged in the opposite direction, as the *benefits* of, for example, drug use are seldom accounted for [3, 8].

For example, SIS aim (successfully, as the evidence indicates) to reduce mortality associated with illicit drug overdoses. As it turns out, many of the feared costs of the programs—increased drug use, concentration of discarded needles, and so forth—have not materialized. On balance, then, SIS provide a net reduction of harms. But this conclusion is reached only by eliminating a number of other harms from consideration. The ‘mixed message’ criticism is often brushed aside, but one can certainly argue that it is harmful for a state to prohibit an activity, selectively enforce that prohibition, and at the same time devote resources to ensuring that individuals that engage in that activity can continue to do so. Most would agree that, if the state decided to employ a special emergency response team to ensure that drunk drivers who got in accidents received prompt medical attention, it would constitute a harmful mixed message. This harm may be difficult or impossible to identify; it may be so diffuse that its impact on any single individual is negligible; it may only apply to those who support the prohibition in the first place; it may be entirely debatable. But it is a harm. The diminution in property values or perceived quality of life of residents or businesses nearby SIS may be a harm that is merely financial or psychological, and impact very few, but these are still harms. Finally, by keeping users of a dangerous drug alive, the state implicitly supports producers and distributors of that drug—that is, it supports and incentivizes violent and antisocial criminals in their continued distribution of a harmful and addictive substance. This may well be due primarily to the fact that these drugs are criminalized, and perhaps decriminalization would reduce the harms they cause; but it is nevertheless clear that the drug and its distributors cause harms, and supporting them by keeping their customers alive is harmful. These are all harms, and if the philosophy of harm reduction is truly utilitarian then they should be counted. But they are not. Why?

There are at least four grounds on which these and other harms may not be counted: theoretical impossibility, practical difficulty, extreme discounting, or irrelevance. We may dismiss the complaint of a parent uncomfortable with explaining to their child that the state prohibits drug use while simultaneously enabling it by claiming that it is not actually a consequence and thus can’t count as a ‘harm;’ by claiming that it is difficult or impossible to adequately quantifiable; by counting it but discounting it to such an extent as to render it irrelevant to any utilitarian calculus; or simply by ignoring it, because we do not see it as a problem.

First, they may also be *impossible* to quantify. If it is the case that some critics of harm reduction are unconcerned with consequences, then perhaps the ‘harm’ of these programs results from the fact that they support behaviors that are *intrinsically* wrong, and cannot be considered in an instrumental manner within a consequentialist framework. This line of thinking is circular: we would rather not count objections to harm reduction as harms, so they must be deontological; and if they are deontological, we do not need to count these objections in a cost/benefit analysis. Moreover, many if not all of the criticisms of harm reduction programs could be expressed in terms of consequences. To use Campbell Brown’s apt term, we could ‘consequentialize’ objections to harm reduction, ‘count’ the costs to individuals or populations of (for example) tacitly condoning illegal behavior, and trade these costs off against the measurable benefits [2]. This may be complex or awkward, but it is not impossible.

A second, more likely reason for the rigging of the calculus of harm reduction may be that many harms are *practically* ‘uncountable.’ Harms that are primarily psychological or political are difficult to quantify, or at least far more difficult than the harms (drug overdoses, mortality, number of discarded needles) that frequently are counted. It may be difficult to predict future possible harms like reduction of property values, and to isolate the contribution of an SIS to that reduction. This is not an unusual dilemma for utilitarians, and is closely related to the familiar aggregation problem—in this case, the difficulty of trading off clear and easily quantifiable harms for a small number of vulnerable individuals against diffuse and difficult-to-quantify harms to a larger and less vulnerable population. However, the practical difficulty of counting some harms does not disqualify them as harms, and a utilitarianism that simply ignores them because they are difficult to count is disingenuous.

What I believe to be the most likely possibility is that many harms remain uncounted simply because harm reduction proponents choose not to count them. It is not that they *can’t* count some harms because it is impossible or prohibitively difficult, it is simply that they *don’t* count them.

Failure to account for certain harms may occur within or outside of a utilitarian framework. The third possibility is that harm reduction proponents really are stringently utilitarian, and *do* account for all possible harms, but simply weight many of them as zero or so infinitesimally small that they do not meaningfully impact a cost/benefit analysis of these programs. It is true that many objections to harm reduction programs are accounted for, and that these feared harms are often overestimated. Supervised injection sites do not increase discarded needles, drug use or initiation; promotion of contraceptive use does not increase sexual activity among students; and so forth. But the fact that the easily quantifiable benefits clearly outweigh the easily quantifiable harms is a happy accident, and does not imply that the unquantified harms will also have been overestimated.

The fourth and most plausible explanation is that harm reduction proponents don’t count the harms proposed by unpalatable criticisms because they dismiss them entirely. For example, many harm reduction proponents (correctly, in my view) reject the ‘mixed messages’ argument because they believe that it relies on an illegitimate characterization of certain behaviors as ‘bad’, and thus unfairly stigmatizes behaviors or people as ‘bad.’ If drugs shouldn’t be illegal in the first place, how can it be harmful for the state to tacitly condone their use and provide clean needles and readily available naloxone? If teenagers having sex isn’t wrong in the first place, how can it be harmful for a school to acknowledge that teenagers might have sex and provide contraceptives to reduce unwanted pregnancies and STI transmission? Clearly, some assessment of right and wrong that takes no account of consequences guides these decisions about what counts as a ‘harm.’

These examples illustrate a more general criticism that utilitarianism cannot stand on its own as a moral theory or guide to action, unless it is united with a theory of the good [19]. Harm reduction proponents would hardly be unique in using a theory of the good to determine which outcomes qualify as harms and benefits, and how to weigh them; this is not a perversion of utilitarianism, but a constituent feature. But that is not what I am arguing. Harm reduction proponents do not incorporate a theory of the good into utilitarian calculations; they employ an alternative moral framework entirely, using utilitarianism only as a convenient, post hoc framing device. Indeed, a recent survey of harm reduction practitioners derived from their responses a set of guiding principles—humanism, pragmatism, individualization of treatment, promotion of autonomy, incrementalism, and accountability without termination—that did not include any mention of utilitarianism, consequences, or cost/benefit calculations [9].

### If not Utilitarianism, Then What?

Contending that a philosophical framework is difficult or impossible to practically implement does not disqualify it as a useful tool to think with, even if it may consign it only to the realm of ideal theory. Utilitarianism is a useful means of encouraging us to dispassionately assess benefits and harms to the greatest extent possible, to acknowledge and confront the potential tradeoffs inherent in any policy or intervention, and to consider the competing claims to benefits and harms of different parties who are impacted—however tenuously—by these policies and interventions. The utilitarian justification of harm reduction is useful insofar as it helps us to confront these questions.

Emphasizing the utilitarian aspects of harm reduction may also be an effective pragmatic strategy for justifying otherwise politically unpopular programs. Yet these contingent justifications are tactical, not essential, and we should not misread the public relations arm of a social movement as its *raison d’être*. Utilitarianism, which implies a dispassionate calculation of benefits and harms across an entire population, does not represent the ideal behind harm reduction programs, nor does it capture the essential quality that separates them from other public programs or policies that also reduce harms: an explicit selection of harms on the basis of moral commitments regarding *who is harmed*.

Above, I demonstrated that harm reduction programs are not actually utilitarian in theory or in practice. Below, I will argue that this is fine, because the last thing in the world that we should do is justify the programs that currently bear the name “harm reduction” on the basis of utility or consequences. Utilitarianism is neither sufficient nor necessary for justifying harm reduction, and is ultimately detrimental.

Utilitarianism is not a *sufficient* moral framework to justify harm reduction, because its practical implementation requires a *prior* choice of which harms and benefits count, and how they must be weighted. As noted above, this is a constituent feature of utilitarianism, which always requires some theory of the good to identify the appropriate harms and benefits. Yet utilitarian defenses of harm reduction programs tend to downplay or ignore these prior choices altogether, while emphasizing the dispassionate calculation. This gets it precisely backward. The substantive reasons for choosing which harms and benefits to count, among which people, and why, should take pride of place in justifications of harm reduction programs.

Utilitarianism is also *unnecessary* as a moral framework to justify harm reduction. Harm reduction programs are not, ultimately, about ‘reduction’ of ‘harms.’ They are about rejecting the punitive Manichaeanism that stigmatizes and admonishes those who engage in ‘bad’ behaviors. They are about rejecting the idea of moral desert, and showing compassion and respect for individuals, regardless of what kind of behavior they engage in. They are about making the lives of the worst-off slightly less terrible. We may argue over what philosophical framework best captures these impulses, but we can agree on one thing: utilitarianism isn’t it.^1^

It is worth asking why interventions rooted in justice and compassion for the worst off have been given the coldly utilitarian name of ‘harm reduction.’ On one hand, it is a pragmatic response to complex problems whose solutions may not materialize any time soon. Rather than waiting for an end to the War on Drugs, or a sea change in attitudes towards sex, we adopt practical programs to make peoples’ lives a little less bad. On the other hand, it might be a recognition of the meanness of our political moment: in a context of social division and skyrocketing inequality, rejection of Manichaeanism and compassion for the worst-off—particularly stigmatized groups like drug users and sex workers—is so unpopular that we are afraid to invoke it even in the context of public health. We feel that we must frame compassion as calculation, interpersonal warmth as impersonal coldness.^2^

If this is the case, if utilitarianism is simply a pragmatic public relations ploy, some might (aptly) wonder, what’s the harm with ‘harm reduction’? Indeed, shouldn’t we capitalize on the success of extant harm reduction programs by extending their logic to other arenas? So long as these programs help without causing meaningful harms, does it matter what we call them? I think that it does.

First, if you live by the utilitarian sword you may also die by it. It so happens that the tradeoffs that have so far been identified have come out in favor of harm reduction programs; but what if they don’t? What if we counted *all* the harms, and found them to outweigh the benefits? What if, for example, provision of free naloxone for drug overdoses increases drug use?^3^ If we rely on utilitarian justifications, then an unfavorable cost/benefit ratio would lead us to abandon a program that we might otherwise maintain on compassionate grounds.

Second, I think that framing compassionate programs as utilitarian ones ultimately contributes to the continued stigmatization of behaviors and dehumanization of the people that they seek to help. Defending compassionate attempts to address the suffering of the worst-off on the utilitarian grounds that they reduce overall harms, rather than the grounds that they are human beings whose suffering must be addressed, diminishes their moral force. We do not justify extraordinary interventions to help children with leukemia, or to rescue a teenage soccer team trapped in a cave, on the grounds that they reduce harm or improve overall utility—for surely they do not. Why then should we justify helping sex workers or injectable drug users on these grounds, unless we already tacitly accept that they do not ‘deserve’ our help in the same way that child cancer patients or teenage soccer players do?

Finally, I think that framing compassionate programs in utilitarian terms unnecessarily, and wrongly, cedes the ‘moral high ground’ to critics. ‘Harm reduction’ is often pitched as the value-neutral, less-controversial label for programs that might be unpopular because they are associated with ‘bad’ behaviors or ‘bad’ people. This might be politically pragmatic, but it is ultimately harmful because it implicitly accepts the designation of these behaviors and people as ‘bad’ rather than rejecting these labels, challenging the moralizing, and offering the positive argument that human beings deserve respect and compassion. Claiming an instrumental ethos for harm reduction invites opponents to claim an intrinsic motivation which in the popular arena reads as “proponents of harm reduction just care about numbers, opponents care about what’s right.” This claim is tendentious, but so long as defenders of compassionate programs embrace instrumentalism it will persist.

The rightness of ‘harm reduction’ programs—and what distinguishes them from other public health interventions—has nothing to do with harm and nothing to do with reduction. Their rightness inheres in rejecting Manichaeanism and moral desert, and respecting and giving priority to the lives of the worst off. It is not and should not be subject to a consequential calculus, and we should not pretend that it is or should be. We should not cede the territory of rightness to the critics; we should instead forcefully proclaim the conviction that helping those who suffer is intrinsically right. ‘Harm reduction’ is a misnomer, and the name should be abandoned sooner rather than later.

## Footnotes

1 Christie et al.[7] argue that virtue ethics provides such a framework. While I agree with many of their substantive claims regarding the centrality of compassion in harm reduction programs, I think they miss a number of aspects of harm reduction that can’t be subsumed within virtue ethics.

2 Though even this might not be enough. One empirical survey of attitudes towards harm reduction found that participants were far more sympathetic to programs that reduced the harms of skateboarding than those that reduced the harms of heroin injection, with ten sex and tobacco use falling in the middle—suggesting that harm reduction may not be particularly effective at reducing social stigma [14].

3 This is the controversial conclusion of a recent study in the US, which found that broadened naloxone access “led to more opioid-related emergency room visits and more opioid-related theft, with no reduction in opioid-related mortality.” [6].
